# Diagnostic and surgical management of the first reported case of bilateral schwannomas of seminal vesicle at a single center

**DOI:** 10.3389/fsurg.2025.1672699

**Published:** 2025-11-20

**Authors:** Tao Ma, Guihua Cao, Jianping Du, Lingfei Wang, Jie Wang

**Affiliations:** 1 Department of Urology, The People’s Hospital of Leshan, Leshan, China; 2 Department of Urology, People’s Hospital of Leshan Wutongqiao District, Leshan, China

**Keywords:** seminal vesicle, schwannoma, urogenital tumor, laparoscopic resection, diagnosis

## Abstract

**Background:**

Schwannomas are benign tumors originating from Schwann cells and are rarely found in the seminal vesicles. Due to their deep pelvic location and nonspecific symptoms, these tumors are often discovered incidentally during imaging or physical examinations. We report a rare case of bilateral schwannomas of seminal vesicle in an asymptomatic 68-year-old male, highlighting the diagnostic and treatment of such uncommon tumors.

**Case presentation:**

This case describes the successful diagnosis and management of bilateral schwannomas of seminal vesicle in an asymptomatic elderly male. Laparoscopic resection achieved complete tumor removal with no perioperative complications. Short-term follow-up showed favorable recovery and no evidence of recurrence.

**Conclusion:**

Schwannomas of seminal vesicle is a rare, slow-growing disease with an insidious onset and low incidence, most commonly affecting middle-aged and elderly men. More than 40% of patients are asymptomatic. The tumor occurs with approximately equal frequency on either side, while bilateral involvement is exceedingly rare. Lesions are predominantly solid, and minimally invasive surgery—either laparoscopic or robotic—is the preferred treatment approach. The overall prognosis is favorable, and to date, no cases of recurrence or malignant transformation have been reported.

## 1 Introduction

**Figure 1:**
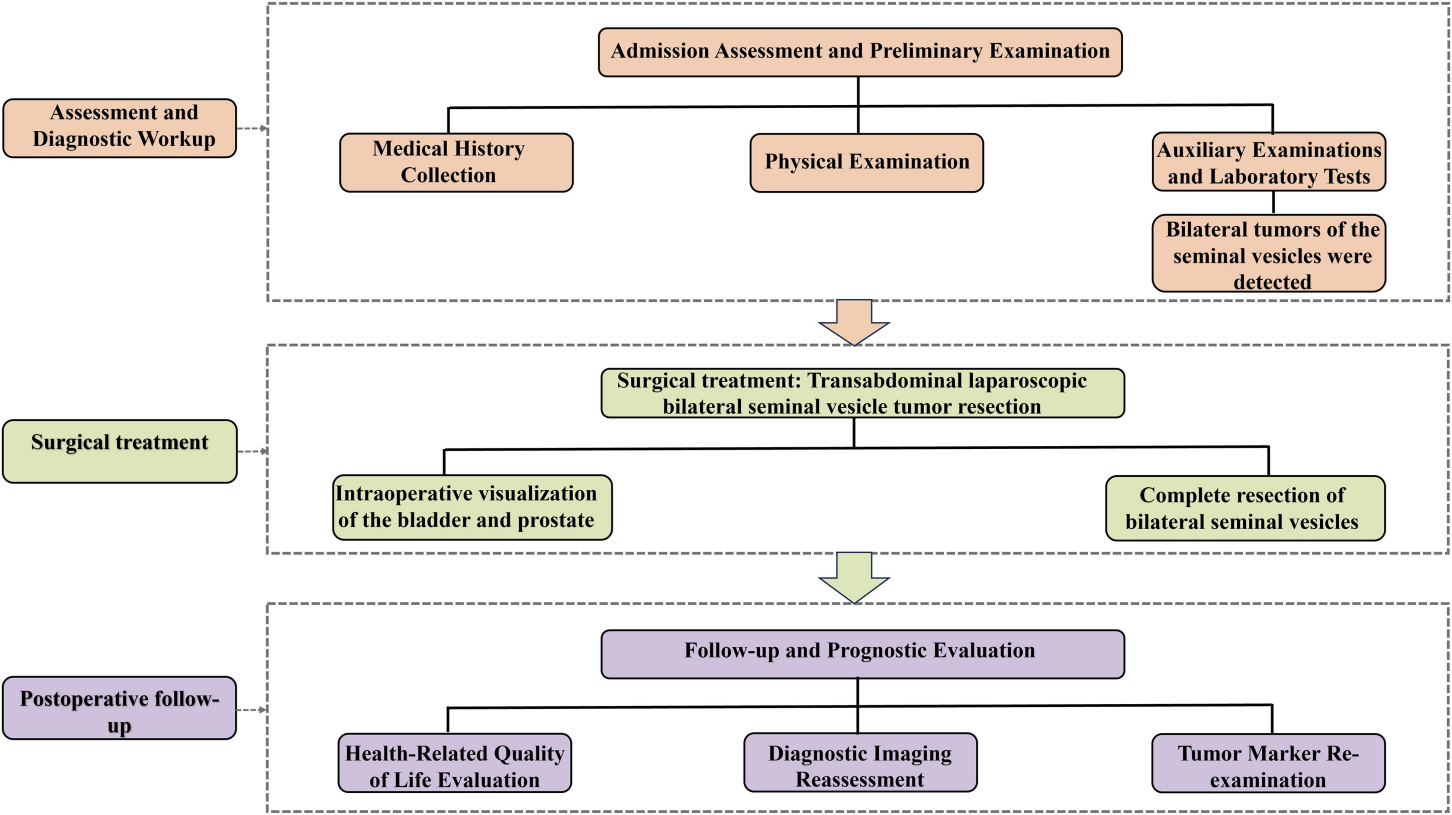
Patient diagnosis and treatment flowchart.

Schwannoma is a tumor originating from Schwann cells, which can occur in peripheral nerves throughout the body. The incidence of schwannomas in the urogenital system, particularly in the seminal vesicles, is extremely rare, and they are typically solitary cases. Bilateral schwannomas of seminal vesicle represents an exceptionally rare case type. As of now, A search of the PubMed database revealed that reports of schwannomas of the seminal vesicle are extremely rare. To date, only 17 cases have been documented in the literature. The clinical characteristics, treatment approaches, and prognosis of these cases are summarized in Table 1. This case report presents an exceptionally rare case of bilateral schwannomas of seminal vesicle (Figure 1).

**Table 1:** Summary of reported cases of schwannomas of seminal vesicle retrieved from PubMed.

No.	Source	Age (years)	Symptoms	Location	Cystic/Solid	Tumor size (cm)	Biopsy performed	Treatment	Prognosis
1	Anjum et al. (1)	42	Haematospermia	Right	Solid	3.4	No	Conservative	No significant change over two years
2	Kushwaha et al. (2)	23	Minor discomfort during defecatio	Left	Solid	7.0	Yes	Laparoscopic surgery	No significant change over three months
3	Chaokamin et al. (3)	67	Moderate LUTS	Right	Cystic	8.1	No	Laparoscopic surgery	No significant change over twenty months
4	Elmer-DeWitt et al. (4)	62	Rectal pain	Right	Solid	5.3	No	Robot-assisted laparoscopy	—
5	Sivashankar et al. (5)	32	LUTS, right loin pain	Bilateral	Cystic	Right:15 Left:5	No	Open surgery	—
6	Shen et al. (6)	40	Nocturia, urinary frequency, intermittent left lower quadrant pain	Left	Solid	3.8	Yes	Robot-assisted laparoscopy	—
7	Mínguez Ojeda et al. (7)	58	Asymptomatic	Left	Solid	3	Yes	Conservative	No significant change over three years
8	Li et al. (8)	54	asymptomatic	Right	Solid	2.4	Yes	Laparoscopic surgery	No significant change over one months
9	Matsuzawa et al. (9)	45	Asymptomatic	Right	Solid	4.8	Yes	Robot-assisted laparoscopy	No significant change over two and a half years
10	Funston et al. (10)	63	Diarrhea and intermittent blood in is stool	Right	Solid	5.0	Yes	Open surgery	—
11	Latchamsetty et al. (11)	48	Right lower quadrant abdominal pain	Right	Solid	2.5	Yes	Open surgery	No significant change over twenty-four months
12	Fievet et al. (12)	36	Asymptomatic	Right	Solid	7.5	Yes	Laparoscopic surgery	No significant change over twelve months
13	Furtado et al. (13)	43	LUTS	Left	Solid	26	No	Open surgery	No significant change over three months
14	Arun et al. (14)	50	Left flank pain	Left	Mixed	13.5	No	Open surgery	No significant change over thirty-four months
15	Huang et al. (15)	55	Asymptomatic	Left	Solid	5.2	Yes	Laparoscopic surgery	No significant change over two months
16	Zhang et al. (16)	48	Asymptomatic	Left	Mixed	8.1	No	Robot-assisted laparoscopy	No significant change over ten months
17	Zhang et al. (17)	42	Asymptomatic	Left	Solid	3.2	Yes	Conservative	No significant change over twenty months
18	Present case	68	Asymptomatic	Bilateral	Solid	8.5	NO	Laparoscopic surgery	No significant change over two months

## 2 Case report

**Figure 2:**
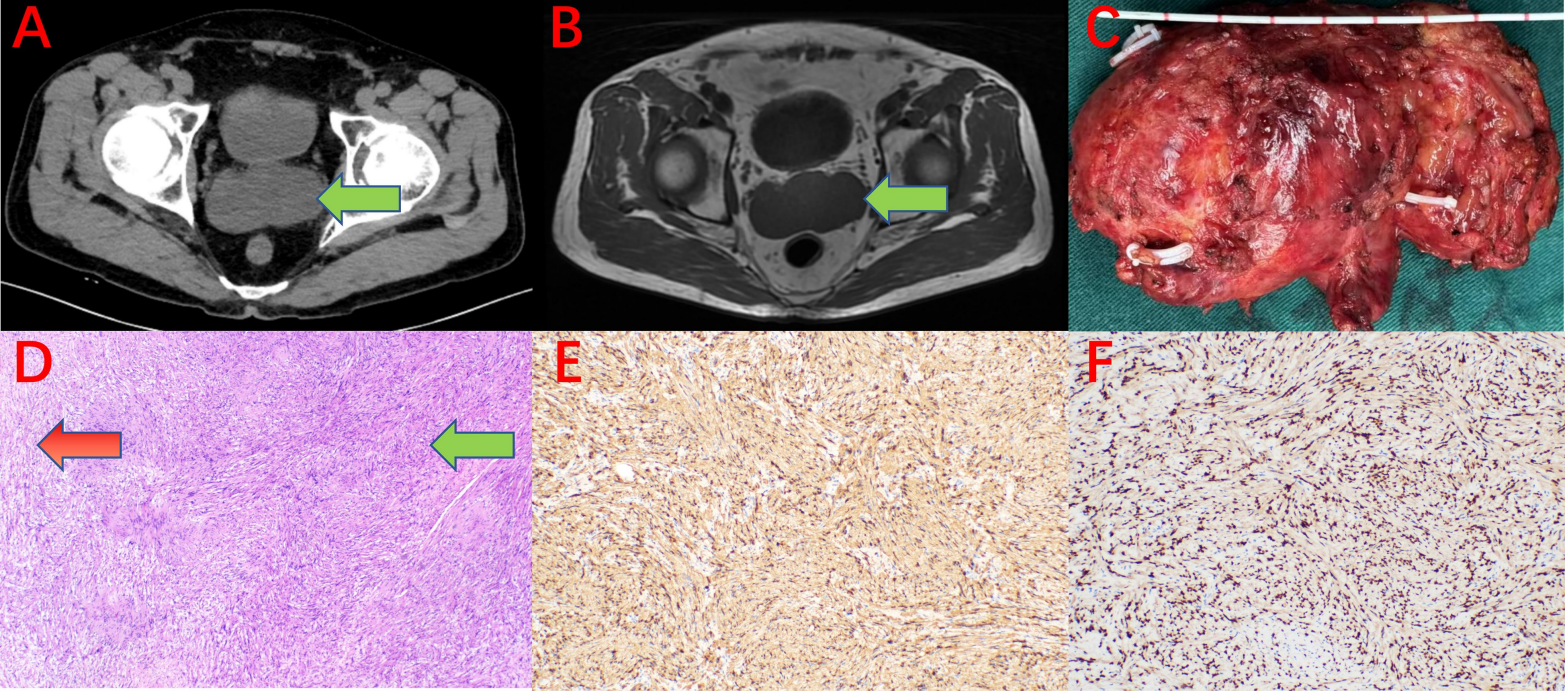
**(A)** CT scan showing bilateral tumors of seminal vesicle (green arrow). **(B)** MRI (T1WI) scan shows bilateral tumors of seminal vesicle (green arrow). **(C)** Surgical specimen (resected bilateral seminal vesicles, vas deferens, and tumor; specimen size: anteroposterior diameter 4.5 cm × transverse diameter 8.5 cm × vertical diameter 5.5 cm). **(D)** Histopathology under microscopy showing Antoni A areas (green arrow) and Antoni B areas (red arrow, HE staining ×40). **(E)** Immunohistochemistry showing S-100 positive expression (×40). **(F)** Immunohistochemistry showing SOX-10 positive expression (×40).

The patient is a 68-year-old married male with a history of good health. He presented to the urology department of our hospital with a 3-day history of a suspected bilateral seminal vesicle mass found during a routine health checkup. The patient reported no hematuria, no perineal or abdominal discomfort, and no difficulty with urination or perineal pain. He also denied any family history of cancer. Upon digital rectal examination, the prostate was enlarged to Grade II, with a firm texture and a shallow central groove. The bilateral seminal vesicles were suspected to be enlarged, with a moderately firm texture and well-defined borders. There was no tenderness or pain upon palpation, and the surface was smooth, with mobility noted. No blood contamination was observed after the glove was removed. Relevant auxiliary examinations were completed, including pelvic computed tomography (CT) (Figure 2A) and magnetic resonance imaging (MRI) (Figure 2B). Given the bilateral nature of the lesions, we considered the possibility of lymphoma or metastatic tumors. After thoroughly informing the patient and their family, we recommended further investigations, including transrectal ultrasound-guided seminal vesicle biopsy and PET-CT, to establish a definitive diagnosis and determine whether the tumor was localized or had distant metastasis. However, due to financial difficulties, the patient and their family strongly declined both the transrectal ultrasound-guided seminal vesicle biopsy and the PET-CT scan. They informed the attending physician that, regardless of whether the tumor was benign or malignant, they insisted on surgical resection. The decision on further treatment would be based on the postoperative pathological report. After completing the relevant imaging examinations and finding no evidence of lesions in other tissues or organs, we considered the patient to have a primary bilateral tumors of seminal vesicl. Preoperative preparation was thorough, and the patient underwent bilateral seminal vesicle resection via a transabdominal laparoscopic approach. During surgery, there was no evidence of tumor invasion into the prostate or bladder, and both seminal vesicles and tumor tissue were completely excised (Figure 2C). All excised specimens were sent to the pathology department for histopathological examination. Histological findings included: 1. The tumor was composed of spindle-shaped cells, showing characteristic whirlpool patterns (Antoni A areas) and loose, mesh-like structures (Antoni B areas, Figure 2D). 2. Immunohistochemical staining showed strong positive expression for S-100 (Figure 2E) and SOX-10 (Figure 2F), with negative staining for CD34, SMAand Desmin, supporting the diagnosis of bilateral schwannomas of seminal vesicle. Postoperative follow-up findings were as follows: The patient returned for evaluation seven months after surgery and reported a good quality of life, with a Karnofsky Performance Status (KPS) score of 90 (see Supplementary Material 1). Mild erectile dysfunction was noted, with an International Index of Erectile Function (IIEF) score of 18 (see Supplementary Material 2). Pelvic MRI and tumor marker assessments revealed no evidence of tumor recurrence or metastasis.

## 3 Discussion

**Figure 3:**
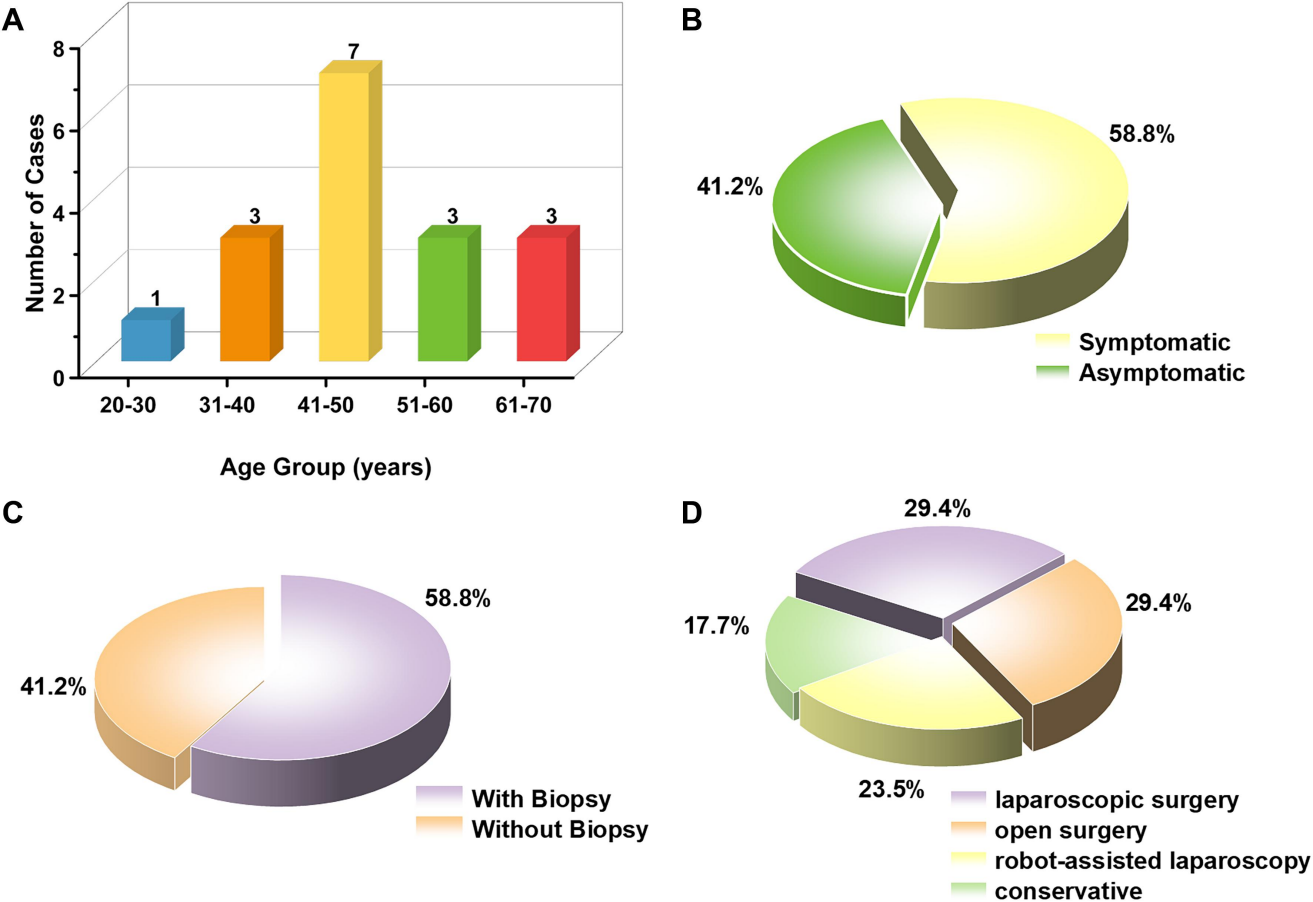
**(A)** Age distribution chart of cases in the published literature. **(B)** The clinical presentation characteristics of cases in the published literature. **(C)** The biopsy rate of cases in the published literature. **(D)** Treatment protocol choices for related cases in the published literature.

Previous case reports have indicated that the age of onset for this condition ranges from 23 to 67 years, with a mean age of approximately 47.5 years, and it predominantly affects middle-aged men (Figure 3A). The clinical manifestations are generally nonspecific: around 59% of patients present with varying degrees of lower urinary tract symptoms, perineal or lower abdominal pain, hematospermia, or defecation difficulties, whereas approximately 41% of cases are discovered incidentally during imaging examinations (Figure 3B). The patient in the present case is 68 years old, representing the oldest case reported to date in the available literature.

Regarding tumor laterality, previous reports have demonstrated a roughly equal incidence between the right and left sides (eight cases each), with only one documented case of bilateral involvement, indicating that bilateral lesions are exceptionally rare. The present case exhibits bilateral lesions, thereby representing a relatively uncommon occurrence in the published literature.

Radiologically, most tumors appear as well-circumscribed solid masses, while a minority present as cystic or mixed lesions. The tumor in the current case is solid, consistent with the majority of previously reported findings.

Tumor size varies considerably, with reported maximum diameters ranging from 2.4 to 26 cm and an average of approximately 7.2 cm. The tumors were generally large, suggesting a highly insidious nature and delayed detection. In this case, the maximum tumor diameter is 8.5 cm—slightly larger than the mean but still within the previously reported range.

Due to the few cases reported in the literature, data for the optimal management of seminal vesicle neoplasms are limited (7). It is generally accepted that benign and asymptomatic tumors can be managed conservatively through regular follow-up examinations. However, for lesions suspected of malignancy or associated with relevant symptoms, surgical intervention remains the preferred treatment option (18). It remains controversial whether schwannomas of the seminal vesicle eventually progress to malignant tumors (19, 20). However, there are currently no clear recommendations regarding the routine use of preoperative biopsy for schwannomas of seminal vesicle (13). Among the 17 previously reported cases, 10 patients (58.8%) underwent preoperative biopsy, while 7 (41.2%) did not (Figure 3C). Among symptomatic patients (*n* = 10), 4 underwent biopsy and 6 did not. Among asymptomatic patients (*n* = 7), 6 underwent biopsy and only 1 did not. Overall, asymptomatic cases were more likely to undergo preoperative biopsy (6/7), whereas symptomatic cases more often proceeded directly to surgery or other interventions (only 4/10 underwent biopsy). Drawing on the ten-year follow-up study by Hajjoff on vestibular schwannomas, approximately 35% of patients who initially underwent conservative management eventually required surgical intervention (21). Based on these findings, we suggest that for patients with schwannomas of seminal vesicle who decline biopsy, direct surgical excision may be considered after a comprehensive assessment of their overall condition.

In this case, the patient refused to undergo ultrasound-guided seminal vesicle biopsy for several reasons. These included limited financial resources and unwillingness to incur additional diagnostic expenses, concerns from the patient and his family about the potential risks of the biopsy procedure, and the patient's strong preference for definitive surgical treatment. In addition, the surgeon's confidence and extensive experience in laparoscopic resection further encouraged both the patient and the medical team to proceed directly with surgical excision rather than pursue further diagnostic evaluations.

In terms of treatment, surgical resection was the primary management approach in previously reported cases, including five cases treated with laparoscopic resection, five with open resection, and four with robot-assisted resection. Additionally, three cases were managed conservatively through observation (Figure 3D). The overall prognosis was favorable, with follow-up durations ranging from several months to several years, and no significant recurrence or malignant transformation reported in any case.

Given the rarity of this tumor type, further multicenter studies are needed to accumulate more cases and optimize treatment strategies.

## 4 Conclusion

Schwannomas of seminal vesicle is a rare, slow-growing disease with an insidious onset and low incidence, most commonly affecting middle-aged and elderly men. More than 40% of patients are asymptomatic. The tumor occurs with approximately equal frequency on either side, while bilateral involvement is exceedingly rare. Lesions are predominantly solid, and minimally invasive surgery—either laparoscopic or robotic—is the preferred treatment approach. The overall prognosis is favorable, and to date, no cases of recurrence or malignant transformation have been reported. The underlying pathogenesis of this disease remains unclear, highlighting the need for further multicenter studies with larger sample sizes to better elucidate its etiology and clinical characteristics.

## Data Availability

The original contributions presented in the study are included in the article/Supplementary Material, further inquiries can be directed to the corresponding author.
