# Supplementary figures and images for: Diagnostic and surgical management of the first reported case of bilateral schwannomas of seminal vesicle at a single center
Source: Front Surg. 2025 Nov 20;12:1672699. doi: 10.3389/fsurg.2025.1672699 (PMC12676284; doi:10.3389/fsurg.2025.1672699)

#
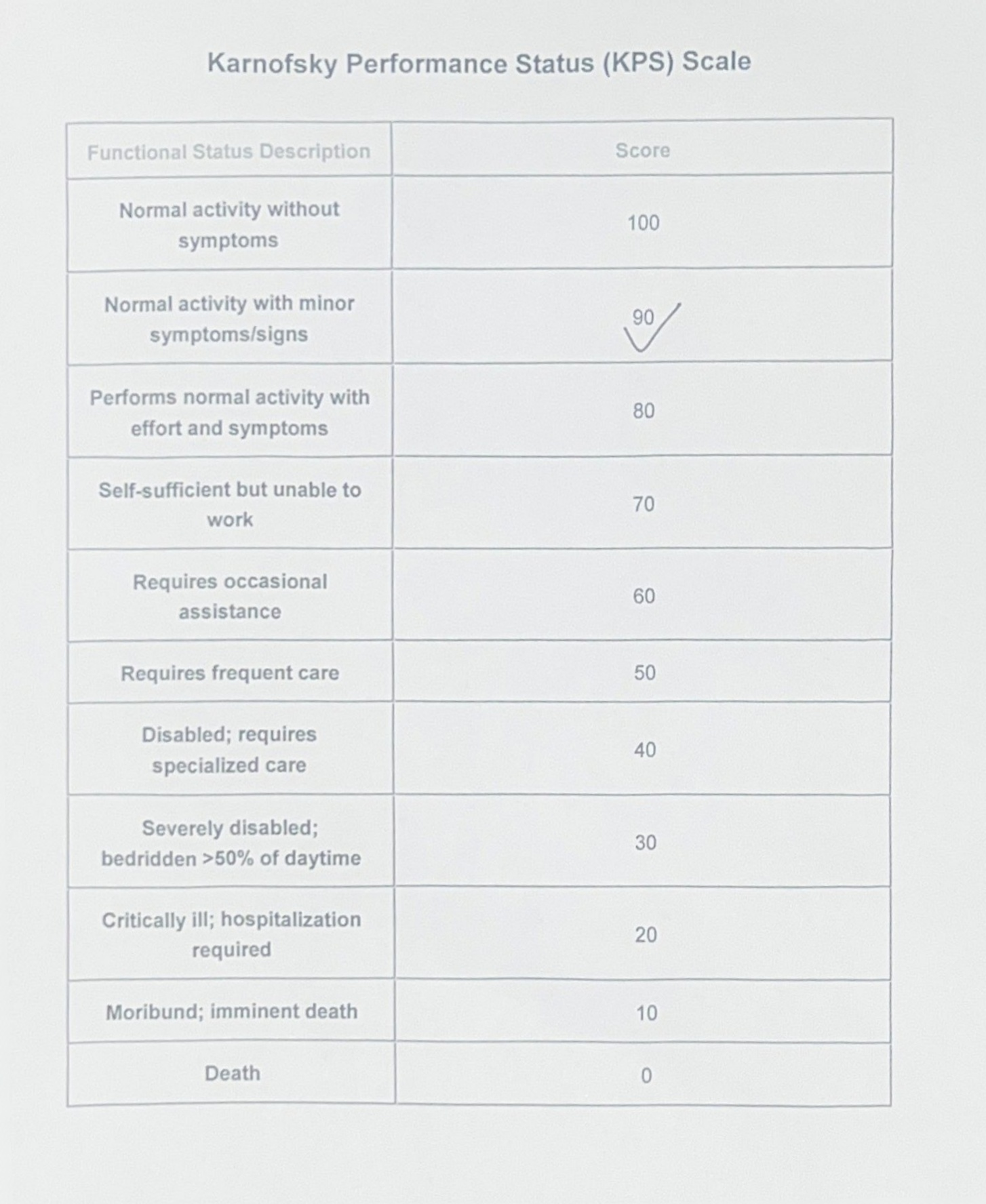

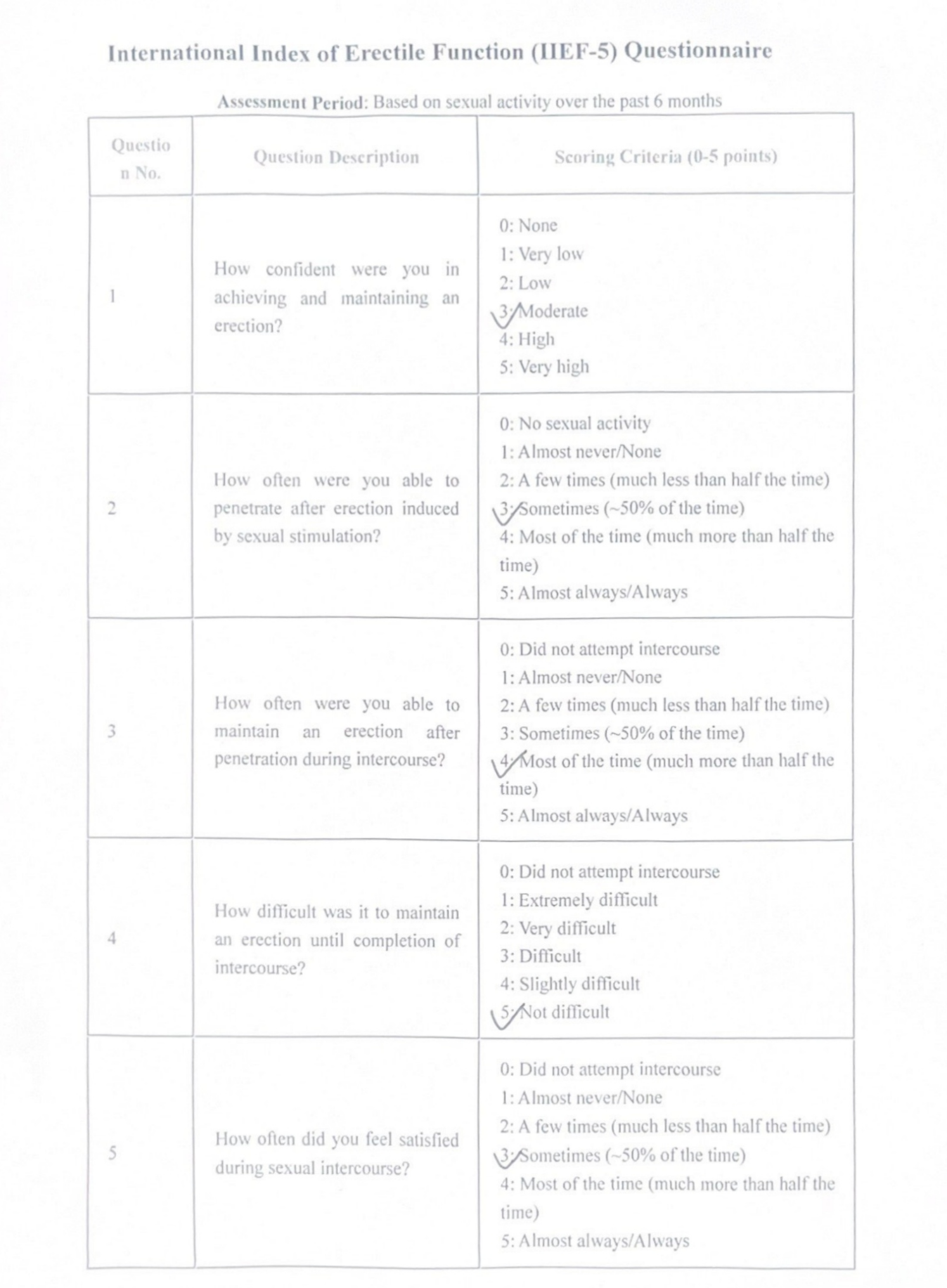

Supplement: Supplementary file 1 [file Datasheet1.docx]
